# Abstract withdrawn

**DOI:** 10.1186/1475-2875-11-S1-P26

**Published:** 2012-10-15

## Abstract withdrawn

